# Critical Assessment of the Chemical Space Covered by LC–HRMS Non-Targeted Analysis

**DOI:** 10.1021/acs.est.3c03606

**Published:** 2023-09-13

**Authors:** Tobias Hulleman, Viktoriia Turkina, Jake W. O’Brien, Aleksandra Chojnacka, Kevin V. Thomas, Saer Samanipour

**Affiliations:** † Van ’t Hoff Institute for Molecular Sciences (HIMS), University of Amsterdam, 1090 GD Amsterdam, The Netherlands; ‡ Queensland Alliance for Environmental Health Sciences (QAEHS), The University of Queensland, 20 Cornwall Street, Woolloongabba, Queensland 4102, Australia; ¶ UvA Data Science Center, University of Amsterdam, 1012 WP Amsterdam, The Netherlands; § Queensland Alliance for Environmental Health Sciences (QAEHS), 20 Cornwall Street, Woolloongabba, Queensland 4102, Australia

**Keywords:** non-targeted analysis, liquid chromatography, high-resolution mass spectrometry, chemicals of emerging concern, chemical space, exposome

## Abstract

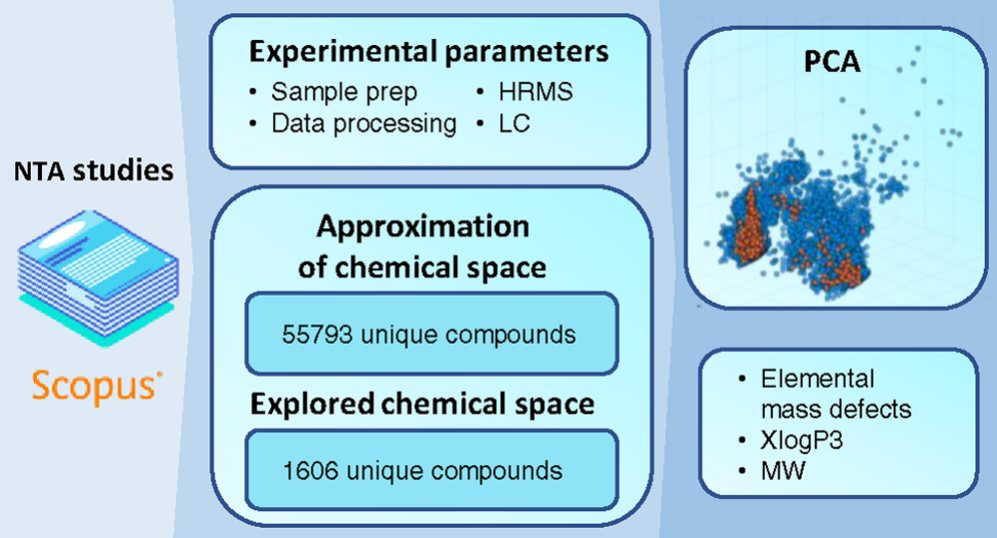

Non-targeted analysis (NTA) has emerged as a valuable approach for the comprehensive monitoring of chemicals of emerging concern (CECs) in the exposome. The NTA approach can theoretically identify compounds with diverse physicochemical properties and sources. Even though they are generic and have a wide scope, non-targeted analysis methods have been shown to have limitations in terms of their coverage of the chemical space, as the number of identified chemicals in each sample is very low (e.g., ≤5%). Investigating the chemical space that is covered by each NTA assay is crucial for understanding the limitations and challenges associated with the workflow, from the experimental methods to the data acquisition and data processing techniques. In this review, we examined recent NTA studies published between 2017 and 2023 that employed liquid chromatography–high-resolution mass spectrometry. The parameters used in each study were documented, and the reported chemicals at confidence levels 1 and 2 were retrieved. The chosen experimental setups and the quality of the reporting were critically evaluated and discussed. Our findings reveal that only around 2% of the estimated chemical space was covered by the NTA studies investigated for this review. Little to no trend was found between the experimental setup and the observed coverage due to the generic and wide scope of the NTA studies. The limited coverage of the chemical space by the reviewed NTA studies highlights the necessity for a more comprehensive approach in the experimental and data processing setups in order to enable the exploration of a broader range of chemical space, with the ultimate goal of protecting human and environmental health. Recommendations for further exploring a wider range of the chemical space are given.

## Introduction

The exposome is the measure of all the exposures, both chemical and nonchemical, of an individual in a lifetime and how those exposures relate to health.^1^ The chemical space of the exposome refers to the chemical space that is relevant to human and environmental exposure.^2−4^ In contrast, chemical space in general refers to all possible organic structures that are plausible from an organic chemistry point of view.^2,5^ Theoretical estimates of such structures suggest that there are around 10^60^ unique structures with molecular weights <500 Da.^5,6^ This theoretical chemical space incorporates both known and unknown unknowns^2,7^ and may include structures that can cause adverse effects depending on their exposure levels. In fact, when looking at the known unknowns (i.e., structures that are recorded in chemical databases that are not initially known to be present in a sample), several of them have been shown to have adverse effects on the environment and human health.^8−11^

Chemical prioritization has been one of the main means for dealing with the diversity of the chemical space in the human and environmental exposome.^3,12,13^ This consists of exploration of the literature for measured chemicals and their properties/toxicities, as well as national/international chemical registries.^14,15^ A combination of predicted properties and toxicities is used to rank chemicals in the databases based on their potential impact on the environment and human health.^16^ Chemicals with a high potential of such impact are considered to be chemicals of emerging concern (CECs).^17,18^ To facilitate chemical prioritization, several databases consisting of chemical structures, their associated physicochemical properties (both measured and predicted), and their biological activities have been made publicly available (e.g., PubChem,^19^ NORMAN databases,^20^ and CompTox^14^). However, most of these known unknowns remain unmeasured in environmental and biological matrices due to the difficulties associated with the inclusion of such a large number of chemicals in routine monitoring programs.^11,13^

Non-targeted analysis (NTA) combined with liquid chromatography–high-resolution mass spectrometry (LC–HRMS) is considered to be one of the most comprehensive methods for the detection and identification of known and unknown unknowns in complex environmental and biological samples.^21,22^ This approach utilizes a generic and wide-scope strategy for the sample preparation and analysis to maximize the coverage of the chemical space of the sample.^2,13,21,23−31^ This typically results in very large and complex data sets (e.g., 5 GB per sample) that must be preprocessed prior to the identification workflow.^31−33^ NTA data processing workflows include several steps, from data conversion to library searches and the confidence assessment of the candidate spectra.^2,23,26−29^ Because of the complexity of such data sets and the sheer size of the chemical databases, NTA workflows are not very sensitive and, thus, do not result in a high percentage of identified chromatographic features.^34,35^ A more sensitive but less comprehensive data processing alternative is suspect screening, in which the chemicals of interest are known prior to the data processing workflow. This approach is more sensitive in terms of the limits of detection, but it is unable to detect unknown unknowns.^20,29,36^ These two strategies are commonly employed together for the screening of complex environmental and biological samples.^23^

Even though the NTA strategy is powerful, it has not been widely accepted within the regulatory framework due to reproducibility issues.^30,34,37^ Recent studies have indicated that small changes in both the experimental (e.g., data-dependent acquisition versus data-independent acquisition) and data processing parameters may result in different outcomes and conclusions.^34,35^ Additionally, a recent study has postulated the potential impact of different experimental parameters on the measured chemical space.^38^ In fact, the aforementioned issues with NTA assays have sparked a debate in the scientific community and have given rise to a new wave of data processing tools development.^25,39,40^ Furthermore, great efforts have been put into better defining the quality control and assurance that are needed for such experiments to be successful in the detection and identification of the known and unknown unknowns in complex environmental samples, thus providing a better understanding of the coverage of the analyzed chemical space.^23,38,41−44^

Several recently published reviews discuss in detail the impact different steps have on chemical space coverage through different experimental approaches.^2,23,26,27^ They cover both data processing and experimental parameters, including the scope of the study, sampling and sample treatment, instrumental conditions, data processing and treatment, and reporting. However, none of these reviews attempted to assess (i.e., quantify) the coverage of the identified chemical space reached by the already conducted NTA environmental studies. Quantification of the coverage of the chemical space using an analytical method is not a trivial task. Theoretically, it can be quantified as the number of identified compounds in the given sample divided by the number of all compounds present in the chemical subspace of the sample. Practically, however, this calculation is impossible, due to the complex chemical nature of samples and the number of unknown constituents. Nevertheless, an investigation of the experimentally explored chemical space is highly relevant, as it would enable researchers to be more aware of the limited coverage of the associated chemical space.

In this review, we aim to quantify the coverage of the identified chemical space in recent environmental studies and investigate the relationship between the selected experimental parameters and the explored chemical space. To quantify the covered chemical space via NTA, we collected all recent studies that performed NTA (not suspect screening) and reported confidence levels 1 and 2 in terms of identification and structures.^45^ Additionally, we limited the scope of this study to semipolar and polar chemicals that are analyzable with liquid chromatography–high-resolution mass spectrometry, thus resulting in a total of 61 papers. As an approximation of the chemical space, the NORMAN SusDat database, which contains around 60K unique chemicals with available PubChem CIDs (compound identification numbers), was used (https://www.NORMAN-network.com/nds/susdat/susdatSearchShow.php). We collected a list of experimental and instrumental parameters, including sample preparation (i.e., storage and extraction conditions), chromatographic separation (e.g., eluents, gradient type, and injection volume), high-resolution mass spectrometry settings (e.g., mass analyzer type, data acquisition mode, and polarity), and data processing workflows (e.g., mass and retention time tolerance, retention time domain alignment, and databases used for the search). We also noted any unreported parameters in order to identify the most commonly omitted settings. Furthermore, we extracted information about the scope of the studies and the analyzed samples.

**Figure 1:**
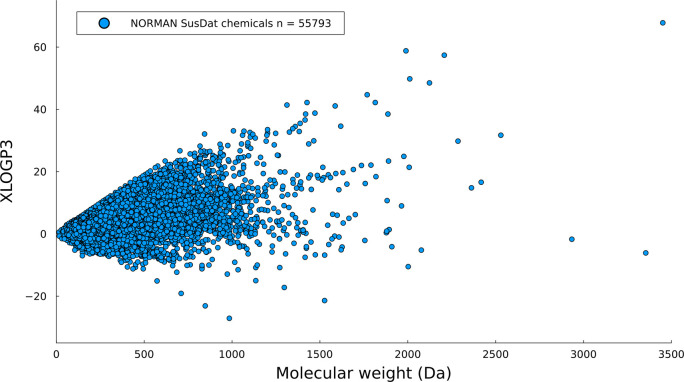
Distribution of all chemicals in the NORMAN SusDat database (*n* = 55 793) based on their molecular weights (Da) and XLOGP3 values.

Finally, we estimated the coverage of the chemical space explored by these recent NTA studies by comparing the structures identified in these studies with the chemical space represented by the compounds in the NORMAN SusDat database, as shown in Figure 1. This figure provides an insight into the range of chemicals that may be present in environmental samples. To our knowledge, this is the first study that “quantifies” the coverage of chemical space via NTA assays.

## Methods

### Selection of NTA Studies

This review is particularly focused on the development of the NTA approach in environmental studies, specifically after discussions regarding reproducibility were initiated.^39^ Thus, we used the citation database Scopus to search for relevant studies published from 2017 to 2023 in the field of non-targeted analysis (NTA) with a focus on environmental science. The search was limited to articles that contained the keywords “non targeted analysis”, “non target analysis”, “untargeted analysis”, “untargeted screening”, or “non-target screening” and excluded articles containing the keywords “metabolomics”, “metabolic”, or “gas chromatography” (GC). This initial search resulted in 377 publications that adhered to the search parameters, which were then manually filtered to include only those that met a specific set of criteria.

The first criterion was that the articles used non-targeted analysis to probe chemicals of emerging concern, preferably in environmental matrices. Second, the publications had to use a non-target workflow. Some articles included the desired keywords in the title or abstract but were actually targeted studies with a very extensive list of target chemicals. For the same reason, the third criterion was that the studies had to have used LC–HRMS for sample analysis. Studies that conducted GC–HRMS were not included in the review, as such studies mainly employ suspect screening rather than non-targeted analysis. Furthermore, the recent development of NTA has been focused on LC rather than GC. Therefore, this review is focused on the coverage of the chemical space by NTA conducted via LC–HRMS. Additionally, direct infusion studies and studies that used rare setups or heavily modified setups were excluded. Finally, review articles and studies that did not perform any identification were excluded, as they did not contribute any additional methods or identified compounds. The search for relevant studies that met these criteria was completed on March 1, 2023, and resulted in the inclusion of 61 studies for this review.^46^

### Collection of Instrumental Parameters

**Figure 2:**
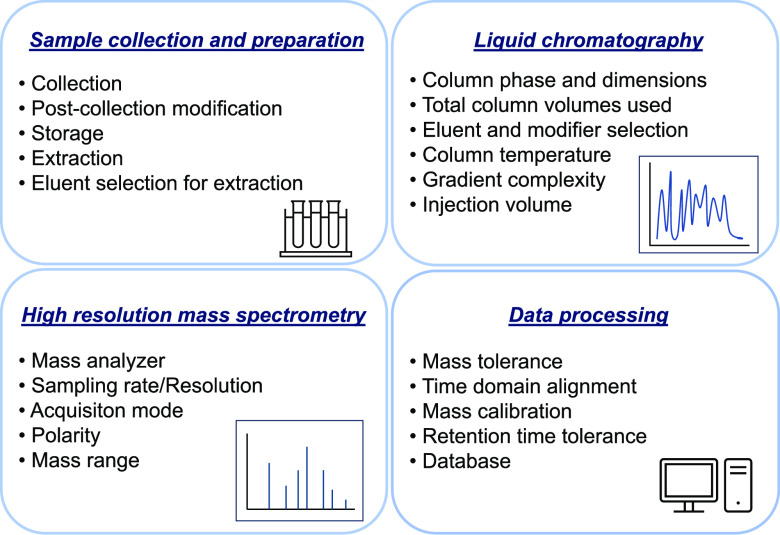
Summary of the main instrumental parameters collected from the reviewed NTA studies.

To capture the impact of each step of the NTA workflow on the chemical space coverage, we extracted specific parameters that were used in the studies we reviewed. Sample preparation, chromatographic separation, data acquisition, and data processing were the four main steps in which parameters were identified. Sample preparation parameters included the sample matrix, storage conditions, prestorage modifications, extraction methods, and extraction conditions (where applicable). Chromatographic separation parameters included the column used, the eluent composition, the gradient complexity, the number of column volumes, the column temperature, and the injection volume. Gradients were classified as linear, semilinear, or complex, based on their complexity. The number of column volumes refers to the volume of solvent that passes through a chromatography column relative to the volume of the column itself. The calculation was performed using eq 1:
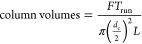
1where *F* is the flow rate (mL/min), *T*_run_ is the total run time of the method (excluding the equilibration time; min), *d*_c_ is the internal diameter of the column (cm), and *L* is the length of the column (cm). HRMS instrumental parameters included the mass analyzer, sampling rate (in the case of quadrupole time-of-flight (Q-TOF)), resolution (in the case of Orbitrap), data acquisition mode, polarity, and mass range. Data processing parameters included mass tolerance, time domain alignment, mass calibration, retention time tolerance, the databases used, and total database size (labeled as small if ≤1000 compounds or large if >1000 compounds). A summary of the collected parameters can be found in Figure 2. We also made note of the parameters that were not reported in order to identify which settings were commonly omitted. Lastly, we gathered information about the scope of the studies. The collected parameters, along with a list of the publications used in this review, are publicly available through the link in ref (46).

### Collection of Reported Structures

To assess the extent of the chemical space coverage by these recent NTA studies, we extracted the reported structures. To ensure the reliability and accuracy of our analysis, we only included structures that were identified with a high level of confidence (i.e., levels 1 and 2 on the Schymanski scale), which are less susceptible to false-positive identifications.^45^ For each compound, the SMILES string, the IUPAC name, and the regular names provided by the authors were extracted. Finally, we excluded articles from our chemical space coverage assessment if the authors did not specify the identification level, did not include the identified compounds in either the article or the Supporting Information, or only reported compounds within their target list.

### Data Processing

The list of the collected compounds was stored in CSV format, and Julia version 1.7 was used to import and process the data. A modified version of the PubChemCrawler.jl package was employed to retrieve chemical data, such as the XLOGP3 and molecular weight (MW) of the compounds, from the PubChem database by using their available identifiers (SMILES, IUPAC, InChIKey, or regular name).^47^ The log*P* values extracted from PubChem are generated using XLOGP3 with an additive model starting from a reference compound.^48^ The retrieved data, along with the collected experimental parameters, were combined into a data set that included the PubChem CIDs of the compounds, their log*P* values, their molecular weights, and the experimental parameters.

For the evaluation of the chemical space coverage, we also calculated elemental mass defects (EMDs) of six elemental ratios (CO, CCl, CN, CS, CF, and CH) for each collected compound and the compounds included in the NORMAN SusDat database.^49^ The EMD values were used to cluster structurally similar compounds together and separate others, as they incorporate structural information and are used to compare compounds based on their elemental composition.^50^ The combination of log*P*, MW, and EMD values was used for principal component analysis (PCA), which is an unsupervised algorithm for dimensional reduction that combines variables into principal components.^51^ This approach is able to identify trends and clusters in the data sets. Prior to the analysis, the data were mean-centered and scaled to keep the initial weight of all variables comparable. PCA was performed using the ScikitLearn.jl Julia package, and in total, three principal components were utilized.

The NORMAN SusDat database was used for approximating the chemical space of environmental samples. While the chemical space comprises both known and unknown compounds, it is practically impossible to include the latter in our approximations. The NORMAN SusDat database includes CECs that either have been detected in various environmental compartments or have been identified as potential CECs, thus providing a comprehensive set of chemicals with a wide coverage of structures and physical and chemical properties.^20^

Finally, the classes of the collected compounds were defined in order to illustrate the frequency of identification of specific classes. To obtain the class of each CEC, the corresponding InChIKey was used to generate information on the superclasses, classes, and subclasses of each compound via ClassyFire. ClassyFire divides a given chemical compound into classes based on its structural features (i.e., functional groups).^52^

## Discussion

In this review, we estimated the coverage of the chemical space of environmental samples by investigating recent NTA studies. To evaluate the impact of the selected workflow parameters on the coverage of chemical space, we collected information on these parameters (e.g., the type of mass analyzer, data acquisition mode, ionization mode, and size of the database used) from the studies.^46^ The identified compounds were categorized into classes, and their relative frequency of occurrence was determined. XLOGP3, MW, and EMD values were used to represent the vastness of the chemical space, which was approximated using the NORMAN SusDat database. PCA was employed to illustrate the coverage of the space of chemicals detected in recent environmental studies.

### Overview of the Studies

In total, 61 studies were collected, with 55 of them having been published since 2020. Only studies that used NTA were included, while those that used screening or targeted approaches that claimed to be untargeted were excluded. This indicates that ≈90% of the reviewed studies were published in the last three years, yielding an average of more than 15 studies per year. In contrast, only six of the selected studies were published during the period from 2017 to 2020. Therefore, the significant increase in the number of such studies in recent years reflects the successful application of NTA workflows in exposome analysis. The scope of these studies varies, with 30 studies focusing on a wide range of chemicals and another 21 studies specifically targeting groups, such as per- and polyfluoroalkyl substances (PFAS), pesticides, pharmaceuticals, and illicit drugs. Such prioritization influences the choice of the experimental setup. The remaining 10 studies focused on NTA workflow development, indicating a growing interest and a need for further advancements in this field.

### Overview of Selected Parameters

#### Sample Collection and Preparation

The collection and preparation of samples in the non-targeted analysis (NTA) workflow can introduce potential sources of loss of chemical information. Issues such as ensuring sample representativeness (e.g., selecting the appropriate grab or passive sampling techniques), addressing potential sample contamination, accounting for matrix effects, optimizing extraction methods for selectivity, and avoiding bias toward specific chemical groups are important considerations in NTA.^2,23,26^ These challenges may impact the accuracy and reliability of the NTA results, potentially affecting the comprehensiveness and quality of the chemical information obtained from the analysis. Therefore, careful attention to sample collection and preparation is essential to minimize potential sources of bias and ensure robust and reliable NTA outcomes.

The majority of the collected studies (67%) analyzed water samples (*n* = 41). Other matrices that were investigated include biota (*n* = 5), dust (*n* = 3), urine (*n* = 3), atmospheric particulate matter (*n* = 2), paper (*n* = 2), serum (*n* = 2), blood (*n* = 1), human hair (*n* = 1), ovarian follicular fluid (*n* = 1), sewage sludge (*n* = 1), snow (*n* = 1), surface soil (*n* = 1), and turtle tissue (*n* = 1).

To prevent microbiological growth, the studies that analyzed water samples reported a conservation step, which involved either adding an acid or storing the sample at a temperature of −20 or 4 °C. Out of the 41 water studies, seven either did not include a step to stop microbiological growth or did not report it. If this step was omitted, it could significantly alter the sample’s final composition when it is eventually analyzed in the laboratory.^53,54^

Around 54% of the publications that analyzed water included a sample filtering step prior to analysis. This step is done to preserve the LC system and column, but it may lead to the loss of the chemicals adsorbed to the particle’s surface. Approximately 67% of the studies included solid phase extraction (SPE) in their sample preparation, out of which 73% used reverse-phase hydrophilic–lipophilic balance (HLB) SPE. However, only 29% of the studies with SPE used acidic or basic modifiers in the extraction eluents. This implies that most of the studies that used only HLB SPE may have potentially been leaving ionizable compounds on the sorbent and may have excluded them from the analysis. The remaining studies employed alternative pretreatment techniques, such as vacuum-assisted evaporation, centrifugation, liquid–liquid extraction (LLE), and ultrasonic extraction, as well as their combination. These choices are mostly dictated by the sample nature/matrix. There were three studies that performed no sample extraction and instead injected the sample directly into the LC–MS with a higher injection volume.^55−57^ While this protocol minimizes sample adulteration and keeps the sampling of chemical space more comprehensive, it can also pose a challenge for detection sensitivity due to the low analyte concentration.^23^

Overall, the sample collection and preparation section was well-reported in the selected studies. However, many of the studies that focused on analyzing a wide range of chemicals did not explore alternative extraction methods to ensure a more comprehensive coverage of the chemical space. This could have resulted in a bias toward specific compounds rather than a more diverse set of chemicals.

#### Liquid Chromatography

Chromatographic separation is employed to minimize sample complexity by spreading analytes across the time axis. This helps reduce ion suppression (matrix effect) and provides additional information (retention time) about the identification of the analytes. The chemistry of the stationary phase, along with the elution conditions, affects the quality of separation and the type of analytes that are retained. Thus, the selection of chromatographic conditions heavily influences the coverage of the chemical space of a sample.^23^

The majority of NTA studies use conventional reverse-phase separation with a generic C18 column. Optimization of the separation involves proper selection of the eluents and modifiers, including suitable elution power and gradient setup, in order to avoid co-elution and an excessive or insufficient retention of chemicals.^58^ A simple linear gradient of an aqueous phase and methanol or acetonitrile from low to high percentage is widely accepted for wide-scope screening. This method has proven its reproducibility across different scopes of studies.^26^ However, this strategy focuses on polar to semipolar compounds, potentially excluding very polar (i.e., log*P* < −2) and very hydrophobic substances (i.e., log*P* > 6) from the comprehensive investigation of the chemical composition of samples.^59^ To cover the polar part of the chemical space, orthogonal methods such as hydrophilic interaction chromatography (HILIC) have become popular, while GC is a widely used technique for hydrophobic, volatile chemicals.^55,60,61^ Finally, to ensure the reproducibility and reliability of the studies, parameters such as injection volume and column temperature should be properly reported.^62^

In this review, more than 90% of the collected studies used a C18 column for the separation, among which almost all were end-capped with a column length of 50 (20%), 100 (49%), or 150 mm (30%). Column diameters were either 2.1 (78% of the studies), 3 (16%), or 2 mm (3%), with the particle diameter being <3.5 μm. Additionally, two different studies reported 4.6 and 0.05 mm column diameters, respectively. Although applying a simple gradient ensures a higher reproducibility of the method, only half of the studies (≈51%) used a linear gradient, while around 32% of the studies used a semilinear gradient and the remaining studies (18%) used a more complex type of gradient.

The median number of column volumes eluted in the studies was 15.9, with an interquartile range of 15.9. The use of a sufficient number of column volumes should ensure the complete elution of most hydrophobic compounds (which have high log*P* and high MW values) and the absence of carryover. The optimal number depends on the stationary phase, the eluent power, and the analytes themselves.^63^ The widely accepted hypothesis is that there is a linear relationship between log*P* and the retention/number of column volumes used. This hypothesis is applied for the reverse-phase mode with comparable C18 selectivity, similar gradients, and eluent composition.^64,65^ However, our results do not indicate the presence of a linear relationship between the number of column volumes and the log*P* of the chemicals, as no clear linear pattern could be identified between these parameters (Figure S1).

The column temperatures that the collected studies used were all slightly above room temperature, which is favorable for repeatability and reproducibility.^62^ Specifically, 31% of the publications used 40 °C, 16% used 35 °C, and 11% used 30 °C. Two studies held the column at 25 °C, one at 20 °C, one at 45 °C, and one at 50 °C. About 29% of the papers did not report the column temperature, which hinders the reproducibility of the experiments.

Finally, 18% of the studies did not report the injection volume used. Injection volume should not have a large effect on the final observed chemical space, as that depends mainly on the extraction method and efficiencies. Nevertheless, the success of a method’s transfer depends on the injection volume. Most of the studies used an injection volume of either 5 (*n* = 17) or 10 μL (*n* = 13), which is adequate when using SPE. The injection volumes of the remaining samples were spread across 1, 3, 4, 7, 20, 100, 140, and 660 μL.

To conclude, despite the increasingly popular discussion about reporting quality,^23,66−68^ the chromatographic separation parameters in the collected studies were not always properly reported. Proper harmonized reporting ensures successful method transfer, whereas inconsistent reporting raises questions about the reproducibility of the study, the reliability of the results, and the possibility of retrospective studies. While the majority of the studies seek to comprehensively investigate the chemical composition of the samples, only approximately 10% employ an alternative to the conventional approach for analyzing samples. Lastly, the hypothetical linear trend between log*P* and retention was not confirmed, indicating the need for more sophisticated strategies for method development and optimization.

#### High-Resolution Mass Spectrometry

Orbitrap and Q-TOF, equipped with electrospray ionization (ESI), are the two most commonly used HRMS instruments in liquid chromatography-based NTA experiments. For complementary analysis, it is recommended that one performs separate experiments in both positive and negative modes.^69^ The mass resolution of Orbitrap mass analyzers is generally higher than that of Q-TOF mass analyzers, but both can provide high-resolution mass spectra (resolution ≥ 30 000).^70^

In Q-TOF, the resolution is determined by the architecture of the mass analyzer,^71^ while for Orbitrap, the resolving power depends on a user-specified resolution. In the case of Orbitrap, the scan speed is directly related to the spectral resolution. However, the increase in the mass resolution is limited by the time required for scanning operations. For Q-TOF, a crucial parameter for data quality is the sampling speed, which is reported as spectra per second in Hz. If the scan rate is too high, fewer ions are sampled, which can lead to a sensitivity issue. Conversely, if the scan rate is too low, fewer data points on the time axis are recorded, potentially causing missed detections of analytes eluting in a narrow time range.^72^

MS/MS spectra for structure elucidation are recorded using either data-dependent acquisition (DDA) or data-independent acquisition (DIA). DDA mode records fragments of preselected precursor ions (which are chosen based on their abundance or via an inclusion list), while DIA mode fragments all precursor ions within a certain mass range. The latter is preferable for comprehensive investigations of complex samples. However, DDA mode is currently the preferred choice in environmental studies, partly due to the limited availability of processing tools for DIA files and also because the DIA experimental setup is not commonly employed with Orbitrap mass analyzers.^26^ Q-TOF analyzers are more commonly used for DIA due to their higher data acquisition rates.

Roughly half of the collected studies (*n* = 31) utilized an Orbitrap mass analyzer, while the other half employed a Q-TOF mass analyzer. However, a significant proportion (approximately 74%) of the studies reported using DDA, which inherently limits their results to predefined ions. The scan rate for the Q-TOF analyzers was mostly set at 4 Hz, although some of the studies operated at lower rates of 3, 2, or 1 Hz. Many of the studies that used Orbitrap analyzers operated at a resolution of 70 000, while some studies used lower resolutions with a minimum of 35 000 or higher resolutions with a maximum of 240 000. Approximately 22% of the studies did not report either the resolution or scan rate that was used.

Less than half of the studies (around 42%) conducted separate experiments in positive and negative modes and utilized multiple injections, different modifiers, and sometimes different columns, which is considered to be a more suitable scenario for achieving a comprehensive coverage of the chemical space. In approximately 30% of the studies, MS was operated only in the positive mode. There were 11 publications where the analysis was reported in both modes, but the details were insufficient for determining whether the experiment was performed simultaneously or separately in both modes. In three other studies, an exclusively negative mode was used to prioritize a specific group of compounds of interest, such as PFAS,^73−75^ deliberately narrowing down the investigated chemical space. Finally, two of the reviewed studies employed simultaneous positive and negative ionization modes with formic acid as a modifier. This approach is not preferable for NTA given that acidic additives are not always optimal for a negative ionization mode. Additionally, the acquired data become extremely complex and lack the quality required for reliable and robust processing.

The selected mass range in the collected studies was between 50–1200 *m*/*z*, which is based on the approximated chemical space covering the largest part. However, some studies set their maximum *m*/*z* to 1000 or lower, which leads to the exclusion of the part of the chemical space with higher MW.

To conclude, despite recent advancements in DIA technology, DDA remained the predominant choice in the reviewed studies. However, the recommended approach for improving the reproducibility and reliability of NTA studies (and for enhancing the coverage of the chemical space in environmental and metabolomics research) is to acquire data in DIA mode for initial screening and then continue with DDA for individual feature identification.^23,76−78^ Finally, in terms of reproducibility, the lack of comprehensively reported information hinders method transfer and therefore warrants actions toward a harmonized reporting strategy.^66−68^

#### Data Processing

Data processing is considered a major bottleneck in NTA workflows. It refers to a series of procedures that starts with data conversion and ends with feature identification.^23^ One of the steps for reliable processing is the mass calibration step, either external or internal. During this step, the measured *m*/*z* values of known structures are compared against theoretical *m*/*z* values. These shifts/correction factors are applied to all mass channels, depending on the instrumental setup. This step ensures the quality of the spectra in terms of accurate mass measurements.^79^ An inadequate mass calibration may result in false-positive and false-negative detections during the identification process.^80^

One of the last steps of CEC identification is to use a database to relate the MS output to a known chemical structure. To proceed with the identification, the experimental data first undergo the following preprocessing steps: data compression, to remove noise and blank peaks; feature detection, to find features in three-dimensional data; componentization, to group together fragments and isotopologues belonging to the same compound; and feature prioritization, to reduce the number of irrelevant features.^81^ Because most of the collected studies used vendor software for the latter four steps, which makes it almost impossible to retrieve the information on the algorithms that were utilized, these parameters cannot be adequately discussed regarding their influence on the coverage of chemical space. For the identification of known unknowns, preprocessed data are compared with chemical databases and matched against references from available spectral libraries by utilizing a combination of features, retention time, accurate mass, and fragmentation patterns.^45^ The mass tolerance is the initial parameter used for the candidates’ list compilation. This parameter, along with the database that is used, heavily affects the results of the candidate search. The number of chemicals included in the databases that were used in the evaluated studies differs from a few hundred structures in in-house libraries^82^ to tens and hundreds of thousands in publicly available libraries,^27^ such as NORMAN,^20^ MassBank,^83^ or PubChem.^19^ These search algorithms result in a set of candidate structures that ultimately must be confirmed via a reference standard and/or an orthogonal method.^45^ The retention tolerance is applied for level 1 confirmation, employing either predicted or measured retention times.^23^

For the transparency and reproducibility of the method, proper reporting of the applied setups for each data processing step is essential. However, a significant portion of the collected studies did not provide sufficient information for one to reproduce the results. Specifically, approximately 39% of the studies did not mention anything about mass calibration, while 25% reported that they performed calibration but did not describe the procedure. Only about 36% included a report on the mass calibration procedure. Additionally, a large number of the papers (43%) did not report whether a retention alignment was performed. While 34% did report that a retention alignment was done, they did not specify the algorithm that was used or provide details about the parameters that were used. The remaining 23% of the publications did report that an algorithm was used, as well as which algorithm was used.

In contrast, the mass tolerance that was applied for the search was reported in almost all of the studies (around 95%). Among these, around 76% used a mass tolerance for the database query of 5 ppm, which is highly common in NTA database search workflows. There were also studies that used a relatively high mass tolerance of 20, 17, or 10 ppm and some studies that used mass tolerances lower than 5 ppm, such as 3, 2, and even 1 ppm. Generally, the studies that used lower mass tolerances for the database search reported a higher resolution of the mass analyzer. However, there was no clear indication of whether the mass tolerance applied to the formula assignment or structural identification. Conversely, retention tolerances had much lower reporting rates, as 45% of the studies did not include this information. The remaining studies used tolerances in a range between 0.1–0.5 min. However, there were a few publications that used a wider tolerance, up to 1.8 min, which may have resulted in a high rate of false positives. Finally, approximately 9% of the studies did not report the databases used, or they referred to the software they used but not to the databases that the software was using. The majority (82%) of the studies used a total database size containing >5000 compounds, while only five studies used databases with <1000 compounds.

The data processing step is one of the main bottlenecks for the NTA approach and thus requires greater attention within the community. Additionally, the reporting quality needs improvement. Furthermore, it was found that around 56% of the identified unique chemicals are available in MassBank EU. This means that roughly 44% of the HRMS spectra that have been acquired for the identified compounds have not been deposited in public databases such as MassBank. In order for NTA to reach its full potential, the expansion of publicly available spectral databases is vital for the improvement of the coverage of the chemical space at the identification step.

### Explored Chemical Space

**Figure 3:**
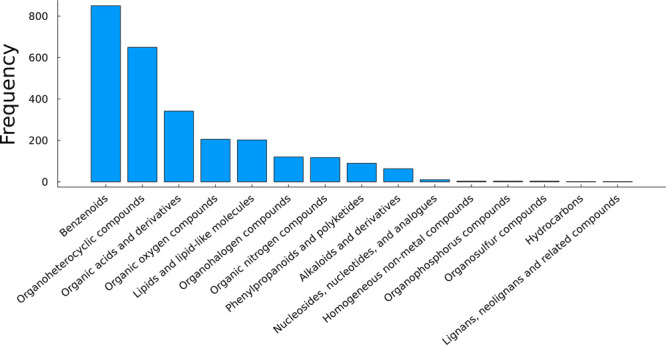
Histogram of all of the classes obtained from the ClassyFire search for the detected CECs in the reviewed studies.

**Figure 4:**
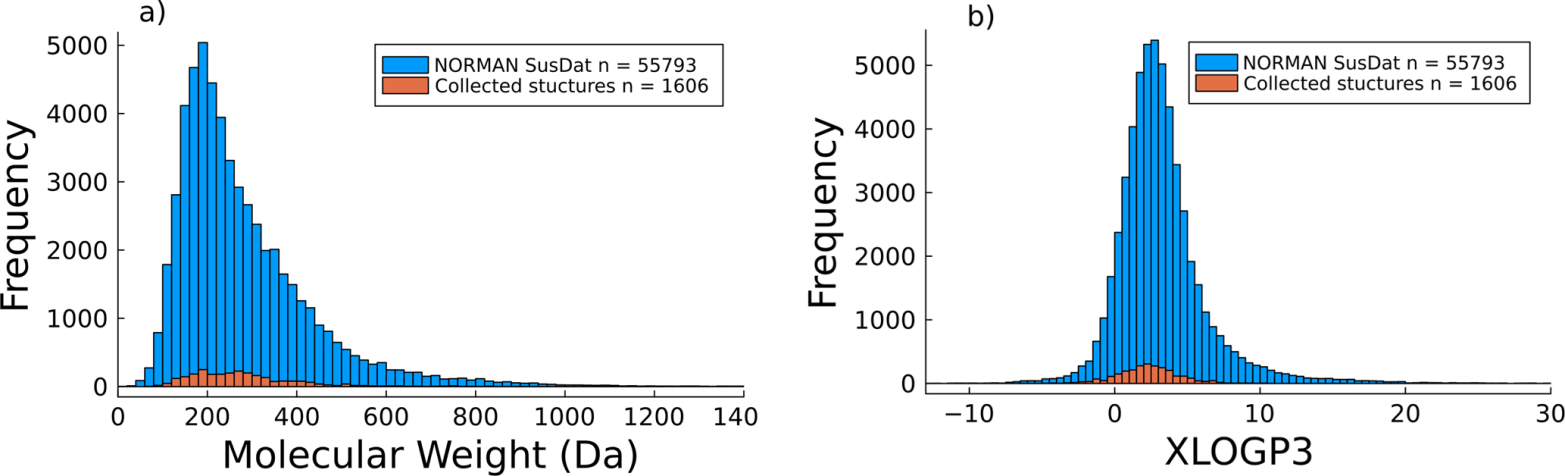
(a) Molecular weight and (b) XLOGP3 distributions for the collected compounds (orange) and the compounds included in the NORMAN SusDat database (blue).

The studies yielded a total of 2657 compounds reported with the identification levels, 1 up to 2b. The contribution to the total number from each study varies between 1 and 370, with a mean and median of 50 and 30, respectively (Figure S2). Among these compounds, 1606 compounds were identified as unique structures, accounting for ≈60% of the total number of retrieved compounds. This finding implies that around half of the overall variety of compounds was detected more than once in various environmental compartments. However, in eight of the studies, there was no report of either the identification level or any identifiers, which hinders the retrieval of the compounds from these studies. The class of each collected CEC was obtained (Figure 3). The most commonly found compounds were benzoids, followed by organocyclic compounds and then organic acids and their derivatives. The latter category, along with organohalogen compounds, contain PFAS, which have been of particular interest in recent years. The median molecular weights of the compounds from SusDat and the compounds collected from the studies were 239 and 261 Da, respectively, and the median XLOGP3 values for the SusDat compounds and the collected compounds from the studies were 3.2 and 2.2, respectively. As seen in the histograms depicted in Figure 4, compounds with the most frequently occurring properties were identified in the recent NTA studies, which can be partially explained by the generalized experimental workflows with reverse-phase C18 columns.

**Figure 5:**
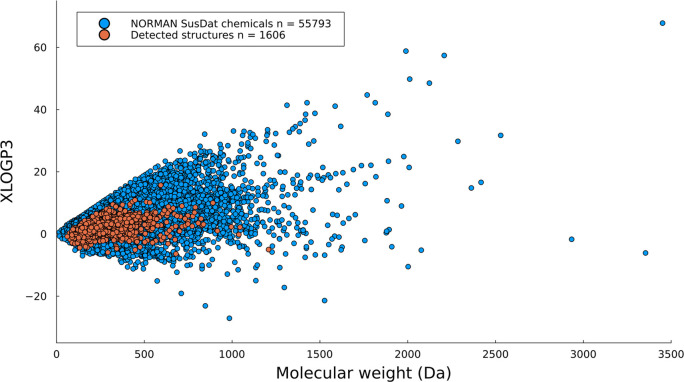
Distribution of all chemicals found in the reviewed articles at levels 1 to 2b (orange), overlaid on top of a distribution of the NORMAN SusDat database chemicals (blue), based on their molecular weights and XLOGP3 values.

As seen in Figure 5, most of the compounds that were detected in the studies clustered closely together, with only a few compounds found farther from this main cluster. The collected compounds were analyzed in relation to their properties and plotted on a chemical space approximation represented by the NORMAN SusDat database. Figure 5 shows the plot in dimensions of MW and XLOGP3, which emphasizes the limited space that is currently explored using current non-targeted analysis workflows. To examine the effect of some of the mass spectrometry parameters that were used on the explored chemical space, all of the compounds were plotted and clustered based on factors such as the mass analyzer type, the data acquisition mode, the ionization mode, and the total database size (Figures S3–S6). However, none of these parameters showed an unambiguous influence on the coverage of the chemical space. It should be noted that the representation using MW and XLOGP3 dimensions does not provide information about the elemental composition of the compounds or their classes, which may result in an over-representation of the covered chemical space. Therefore, it is important to consider other parameters beyond MW and XLOGP3 when evaluating the coverage of the chemical space by the collected structures.

**Figure 6:**
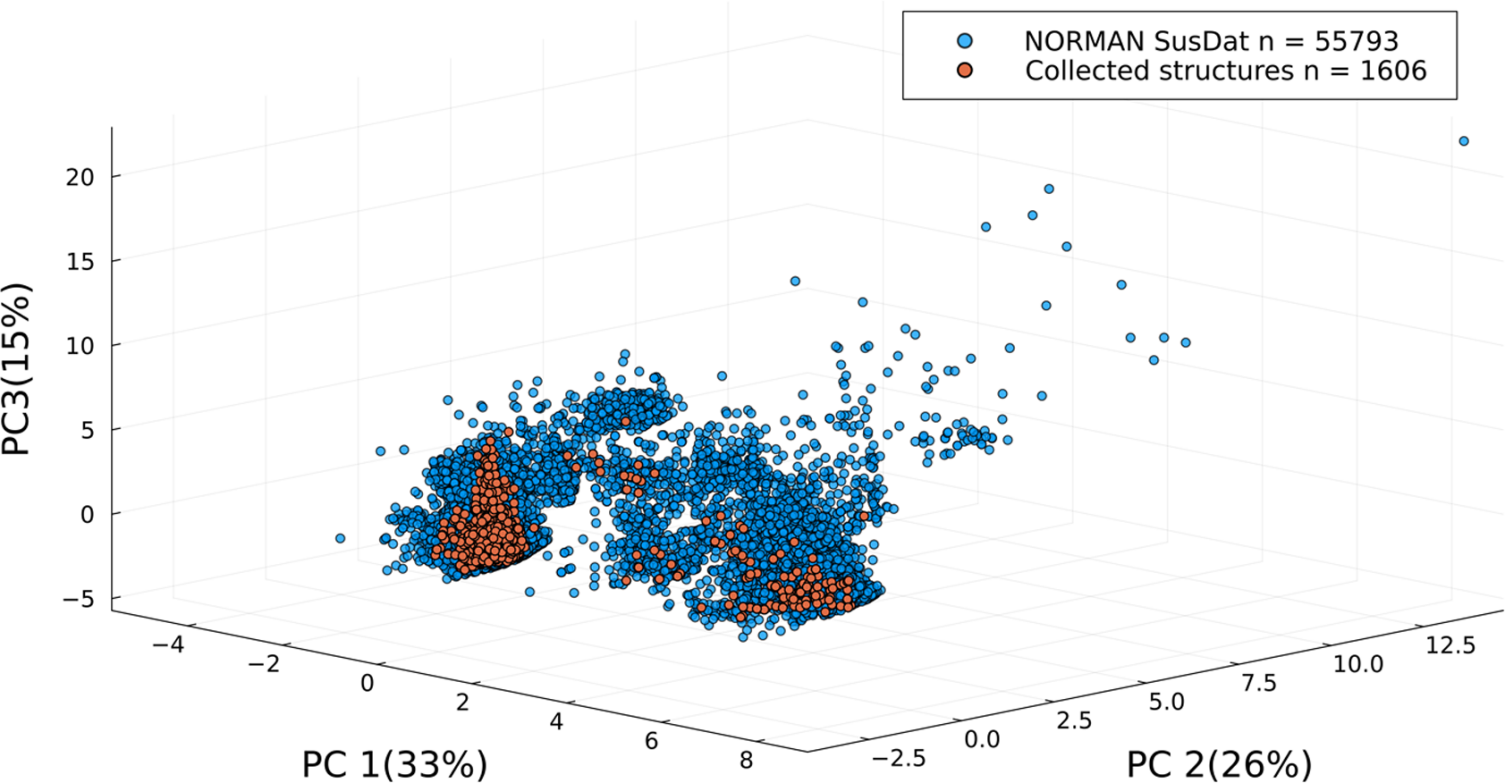
PCA score plot of the three principal components of the NORMAN SusDat database (blue) and the collected structures (orange).

The PCA score plot in Figure 6 reveals that many regions of the chemical space are unexplored. PCA was applied to the data set that combined the collected compounds with the compounds from the NORMAN SusDat database, with MW, XLOGP3, and EMD acting as the input variables. The first two principal components in the analysis were found to be primarily influenced by the EMDs associated with the compounds containing chlorine (Cl), fluorine (F), cyanide (CN), and sulfur (S). These EMDs represent the high variability in the elemental composition of these compounds and were identified as the most important variable in the PCA. This indicates that fewer compounds in the data set contain halogens, nitrogen (N), and sulfur, while hydrogen (H), which is present in nearly every compound, does not contribute significantly to the variability in the data. The third principal component is primarily influenced by MW and XLOGP3 (Figure S7). In total, the first three principal components explain 74% of the variance (Figure S8). In Figures S9–S11, the coverage of chemical space by different compound classes is displayed. Figure S11 specifically highlights the coverage by organic acids and their derivatives, as well as organohalogen compounds. The majority of PFAS, though not exclusively, fall into these classes. These figures reveal that the distribution of compound classes across the chemical space is not homogeneous, suggesting an over-representation of certain classes of compounds. This observation can be attributed to the prior prioritization of specific classes, which may bias the identification toward those classes of compounds.

Overall, only around 2% of the estimated chemical space was covered by the NTA studies investigated in this review. Coverage was defined as the number of unique structures retrieved from the reviewed studies versus the number of structures in the NORMAN SusDat database, which acted as an approximation of the chemical space. We used the NORMAN SusDat database as an approximation of chemical space because it contains a set of highly relevant and curated structures for the environmental and human exposome. It should be noted that this is a small subspace of the total chemical space and serves as a means of approximation for the true chemical space of the exposome. No clear relationship between the experimental conditions and the coverage of the chemical space was discovered, which may indicate that the experimental approaches that were used were generic enough for the NTA assays. Conversely, this may have been caused by the lack of detailed and standardized reporting of the experimental conditions. Therefore, a more rigorous investigation of the parameters and the standardization of reporting criteria must be designed and performed. Although the most widely accepted properties of compounds, such as their log*P* value and MW, are widely used while discussing chemical space,^23^ in this study we showed that they may not be the most relevant markers for assessing the coverage of the chemical space. Finally, such a low coverage emphasizes the need for more comprehensive approaches to experimental and data processing workflows in order to explore a broader range of the chemical space and ultimately protect human and environmental health.

## Recommendations and Outlook

Despite the ability of NTA assays to provide holistic information about the chemical composition of samples, their true coverage of the chemical space has not been investigated. Furthermore, NTA studies suffer from issues related to their reproducibility due to the complexity of both the experimental and computational approaches that are employed in NTA assays. One of the main issues that affects the reproducibility of NTA assays is the lack of standardization for the reporting criteria (including the experimental conditions). Our detailed investigation of previously published NTA studies further suggests the need for such criteria. Minimum accepted experimental criteria and data processing parameters should be reported to ensure transparency and reliability of the results. The utilization of harmonized reporting tools, such as BP4NTA SRT or NORMAN suspect screening reporting tools, can help the reproducibility and transparency of future NTA studies.^68,77,78^ This will potentially lead to the acceptance of the NTA approach by regulatory bodies.

The potential coverage of the chemical space should be assessed during the design of the experimental setup. Most of the recent studies we reviewed used experimental setups that were based on conventional workflows, including using HLB SPE for sample preparation, reverse-phase separation with C18 columns, and DDA mode, and did not consider alternative approaches. The best practice would be to apply alternative extraction methods, implement orthogonal techniques (e.g., reverse-phase LC and HILIC), use DIA mode as the first screening approach, and apply reliable/robust data processing tools, preferably those that are open source/access. For the identification part of the workflow, sharing the experimental mass spectra of the identified compounds and their acquisition conditions is vital for the progression of the community. Additionally, archiving the raw data in public repositories for both retrospective analysis and data processing tool development is highly essential.

To the best of our knowledge, no other study has evaluated the coverage of the chemical space via NTA studies in such detail. However, due to the lack of standardized reporting criteria, the direct impact of different experimental choices on the covered chemical space could not be established. Additionally, our study was limited to the works published after 2017, and we included only studies with clear level 1 and 2 identification reporting. Furthermore, we excluded suspect screening studies, which may have resulted in an underestimation of the coverage of the NTA studies. However, our study, although it was limited, clearly shows the shortcomings of the current NTA practices and the need for further development in different areas, including the experimental setup.

## Data Availability

The information retrieved in this study can be found at 10.5281/zenodo.8249216. References to the reviewed studies and collected experimental parameters are in the Excel file titled, “Tables with parameters .xlsx”. The script that was used to perform the calculations is available at https://github.com//tobihul//Code-for-Critical-assessment-of-covered-chemical-space-with-LC-HRMS-non-targeted-analysis. The PubChemCrawler.jl package that was used is available at https://github.com/JuliaHealth/PubChemCrawler.jl.

## Supporting Information Available

Figures S1–S11, which show the relationship between the experimental parameters and the covered chemical space (PDF)
