# Supplementary material for: Critical Assessment of the Chemical Space Covered by LC–HRMS Non-Targeted Analysis
Source: Environ Sci Technol. 2023 Sep 13;57(38):14101–12. doi: 10.1021/acs.est.3c03606 (PMC10537454; doi:10.1021/acs.est.3c03606)
Supplement: Supplementary file 1 — es3c03606_si_001.pdf [file es3c03606_si_001.pdf]

Supporting information for: Critical assessment of the chemical space covered by LC-HRMS non-targeted analysis

Tobias Hulleman,[†,‖] Viktoriia Turkina,[*,†,‖] Jake W. O'Brien,[‡,†] Aleksandra Chojnacka,[†] Kevin V. Thomas,[‡] and Saer Samanipour[*,†,¶,§]

†*Van 't Hoff Institute for Molecular Sciences (HIMS), University of Amsterdam, 1090 GD, Amsterdam, the Netherlands*

‡*Queensland Alliance for Environmental Health Sciences (QAEHS), The University of Queensland, 20 Cornwall Street, Woolloongabba, QLD, 4102, Australia*

¶*UvA Data Science Center, University of Amsterdam, Amsterdam*

§*Queensland Alliance for Environmental Health Sciences (QAEHS), 20 Cornwall Street, Woolloongabba, QLD, 4102, Australia*

‖*Contributed equally to this work*

E-mail: v.turkina@uva.nl; s.samanipour@uva.nl

List of Figures

S1 Compounds identified in all papers reviewed plotted according to their MW, XLOGP3 and on a scale based on column volumes eluted during liquid chromatography analysis . . . S3

S2 Number of compounds identified in each of the selected papers from highest to lowest . . . S4

S3 Compounds identified in all papers reviewed plotted according to their MW, XLOGP3 and grouped based on the mass analyzer used . . . S4

S4 Compounds identified in all papers reviewed plotted according to their MW, XLOGP3 and grouped based on the data acquisition mode . . . S5

S5 Compounds identified in all papers reviewed plotted according to their MW, XLOGP3 and grouped based on the polarity used in the MS . . . S5

S6 Compounds identified in all papers reviewed plotted according to their MW, XLOGP3 and grouped based on the size of the database used . . . S6

S7 Loading of each variable for every PC of the model . . . S7

S8 Explained variance of the PCA model depending on the number of principle components used . . . S7

S9 PCA of detected CECs grouped by compounds class extracted from ClassyFire S8

S10 PCA of detected CECs and NORMAN SusDat chemicals with the organohalogen compounds highlighted . . . S8

S11 PCA of detected CECs and NORMAN SusDat chemicals with the organohalogen compounds and organic acids and derivatives highlighted . . . S9

1 Exploration of Chemical Space

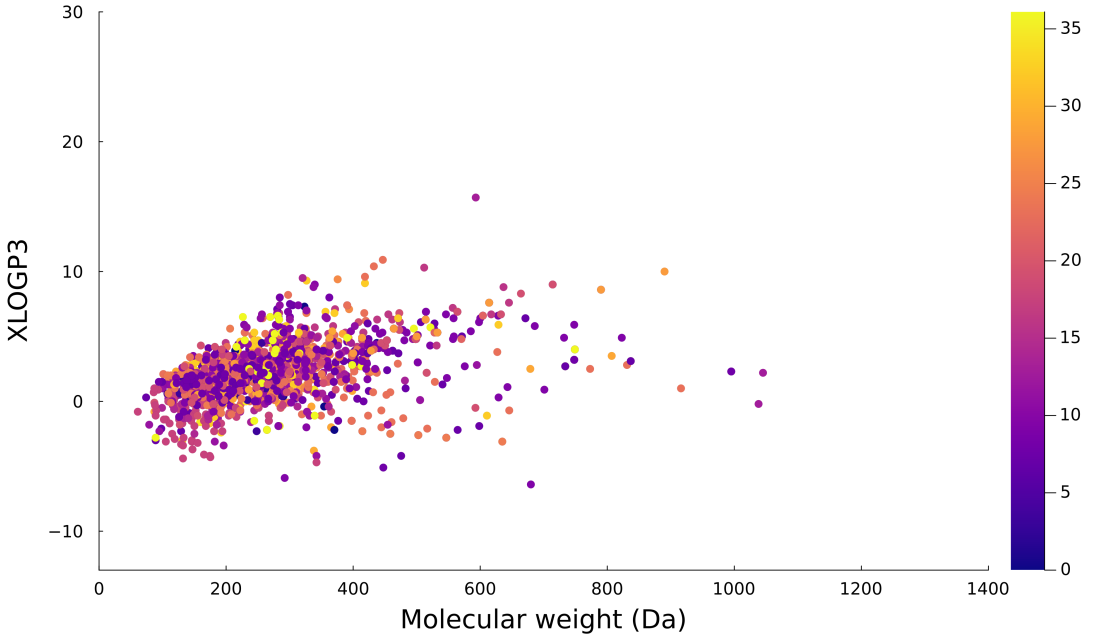

Figure S1: Compounds identified in all papers reviewed plotted according to their MW, XLOGP3 and on a scale based on column volumes eluted during liquid chromatography analysis

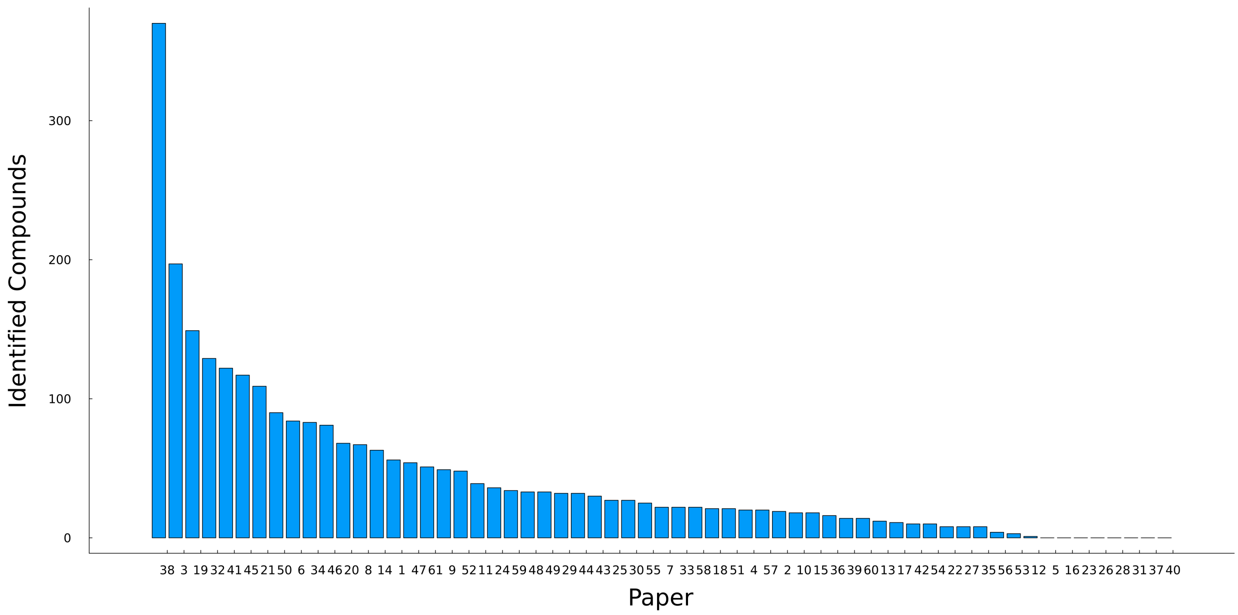

Figure S2: Number of compounds identified in each of the selected papers from highest to lowest

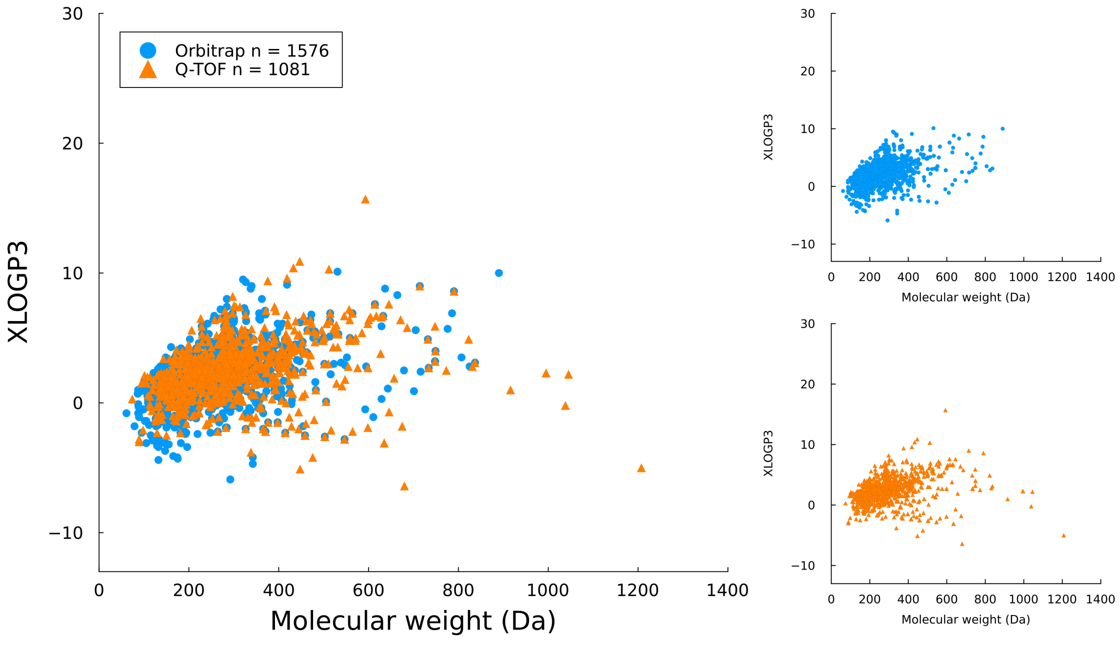

Figure S3: Compounds identified in all papers reviewed plotted according to their MW, XLOGP3 and grouped based on the mass analyzer used

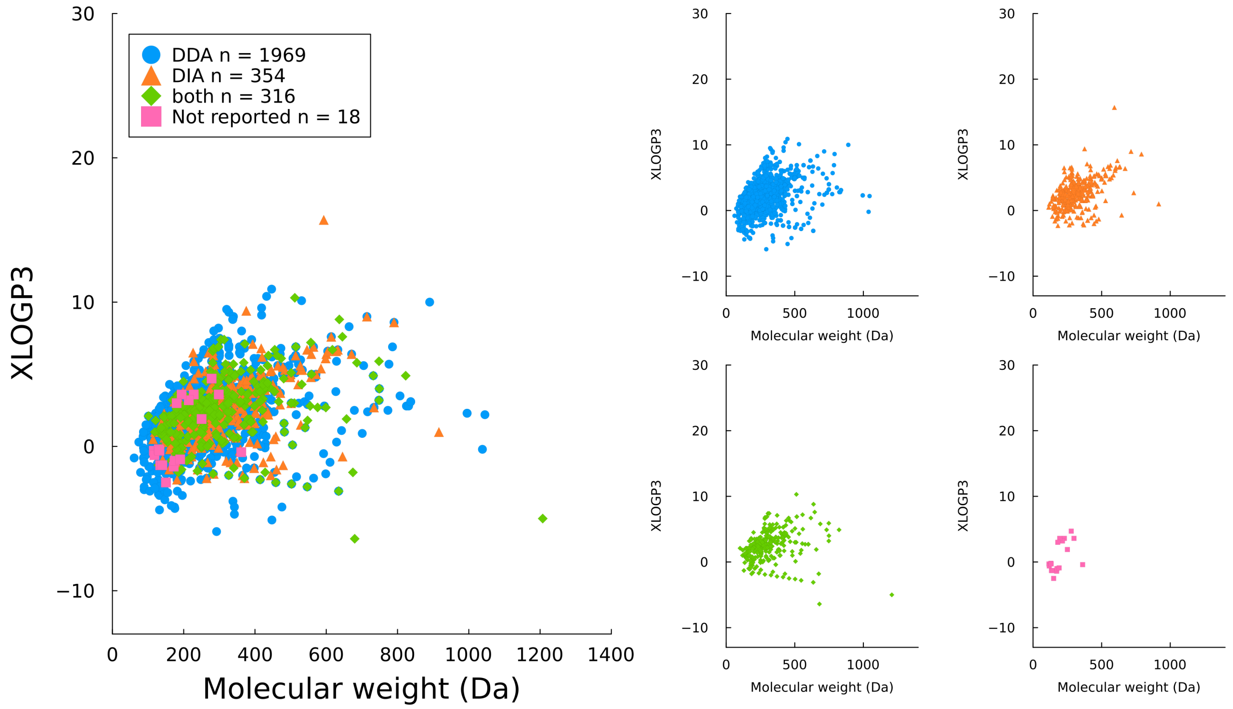

Figure S4: Compounds identified in all papers reviewed plotted according to their MW, XLOGP3 and grouped based on the data acquisition mode

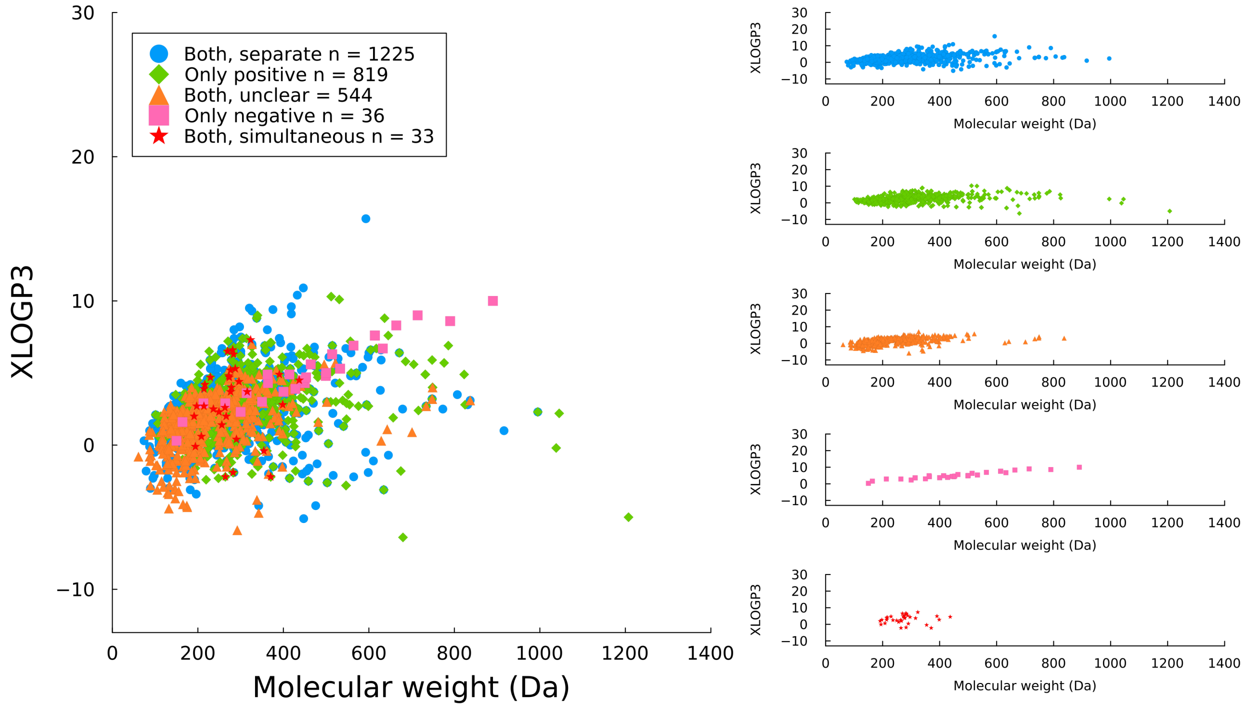

Figure S5: Compounds identified in all papers reviewed plotted according to their MW, XLOGP3 and grouped based on the polarity used in the MS

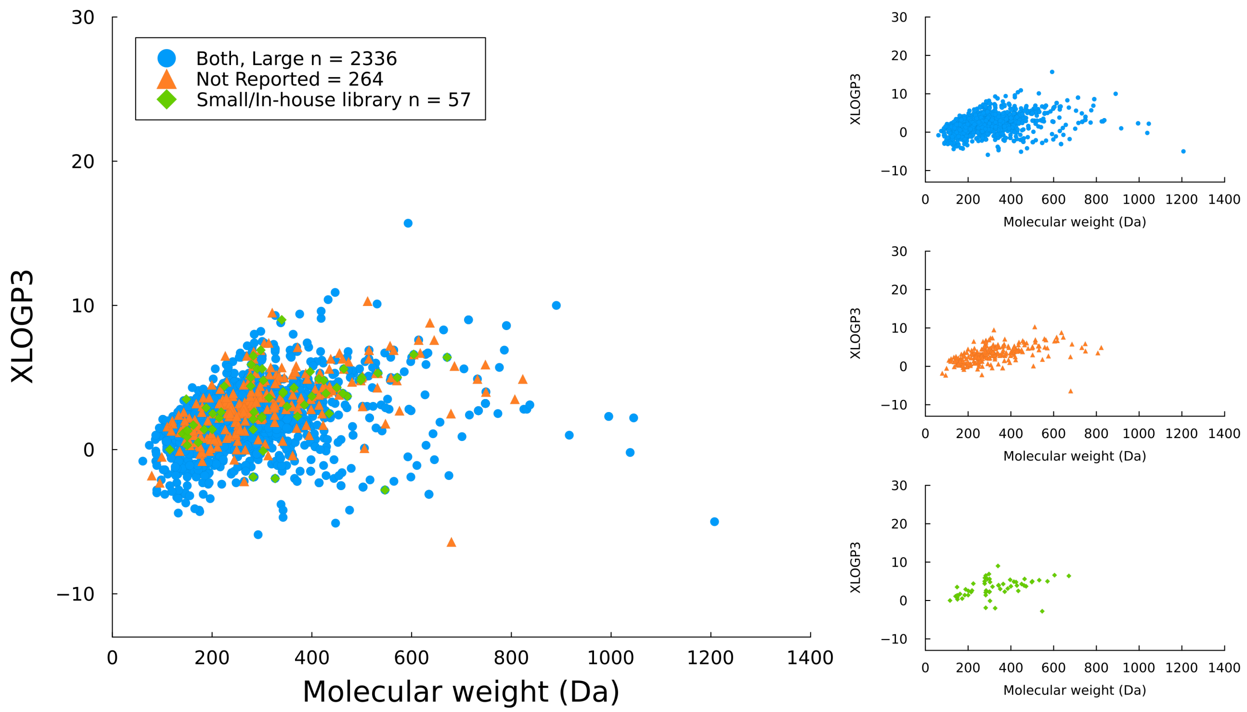

Figure S6: Compounds identified in all papers reviewed plotted according to their MW, XLOGP3 and grouped based on the size of the database used

2 Principle component analysis

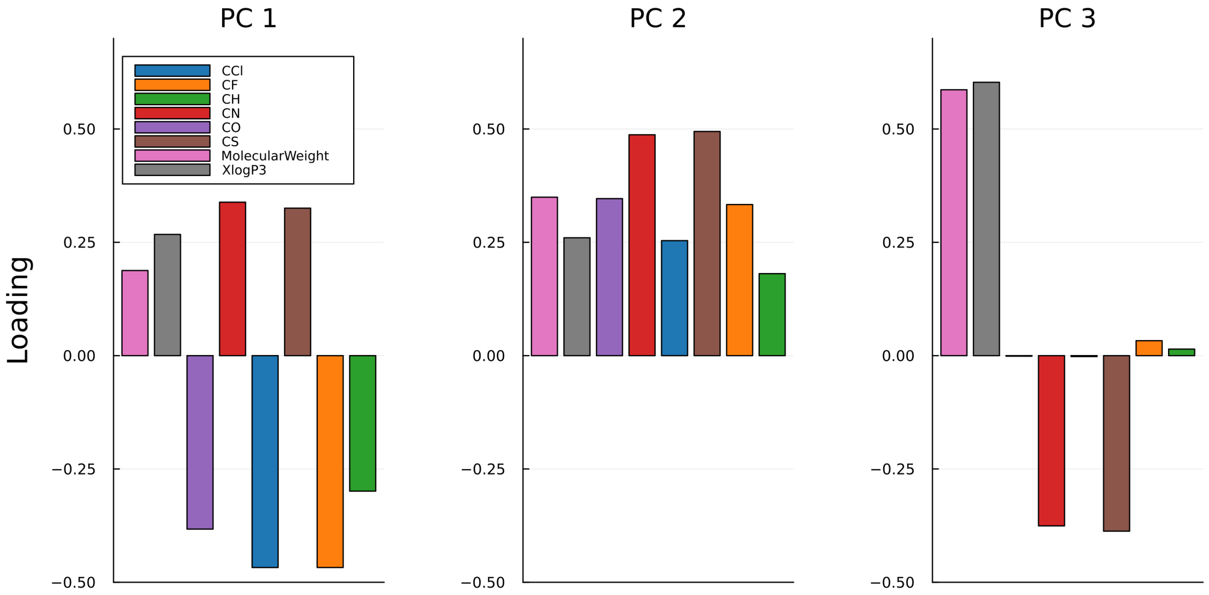

Figure S7: Loading of each variable for every PC of the model

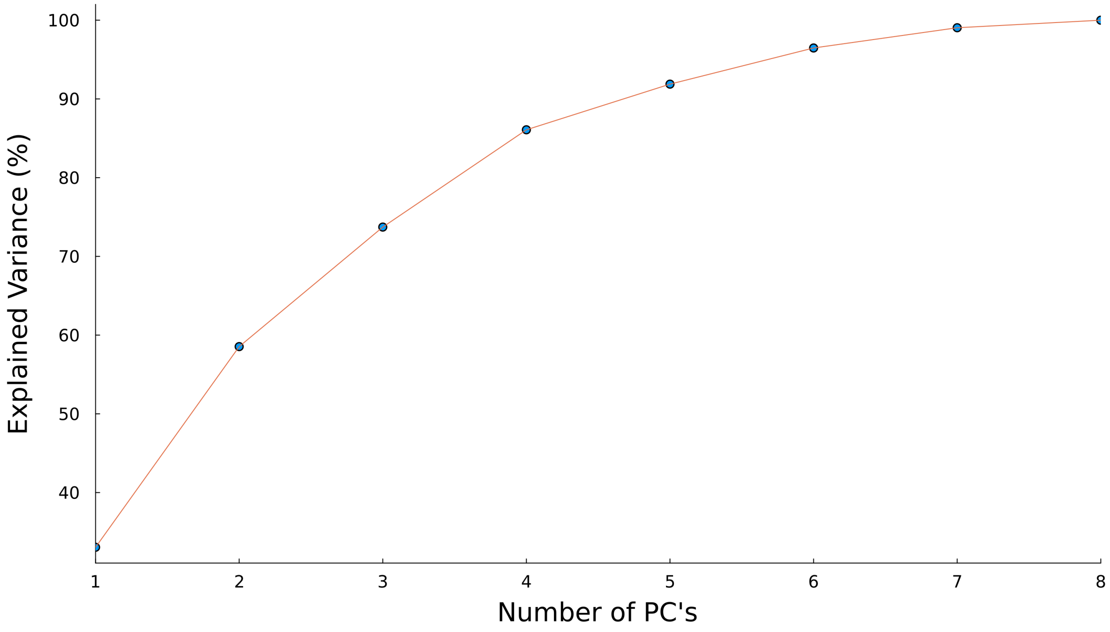

Figure S8: Explained variance of the PCA model depending on the number of principle components used

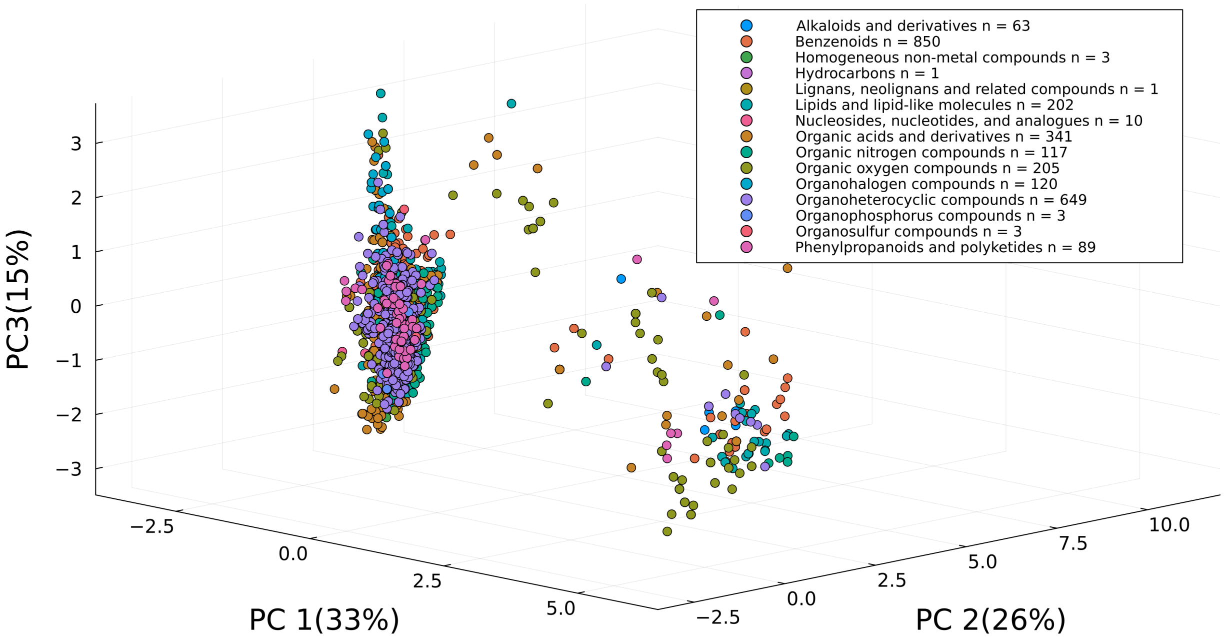

Figure S9: PCA of detected CECs grouped by compounds class extracted from ClassyFire

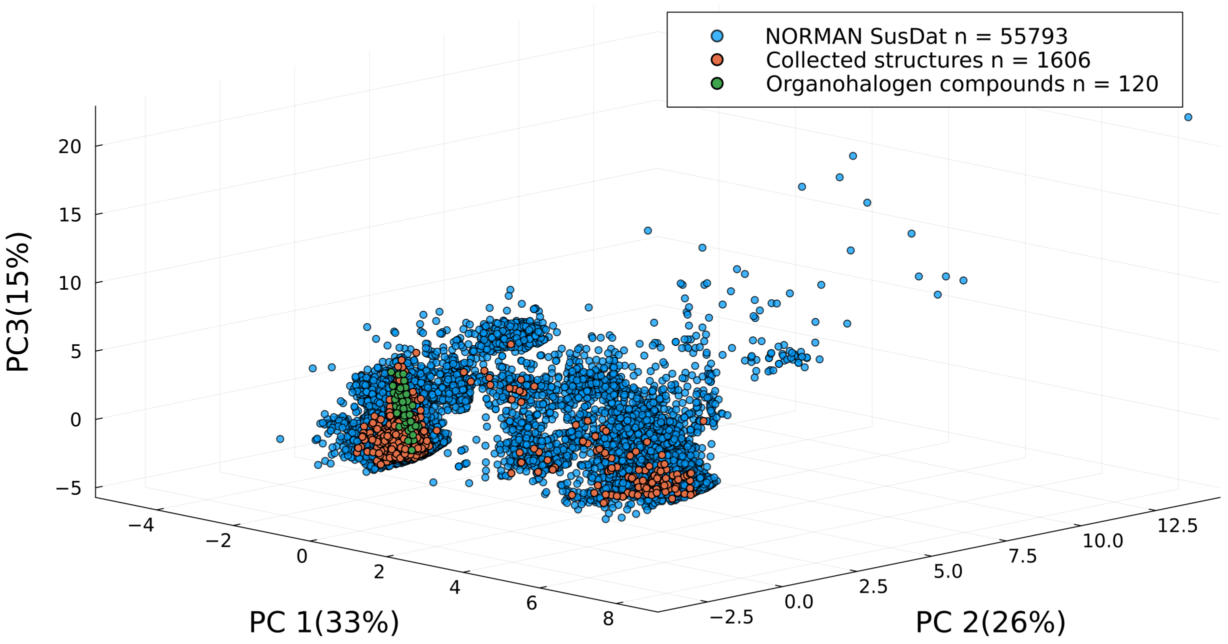

Figure S10: PCA of detected CECs and NORMAN SusDat chemicals with the organohalogen compounds highlighted

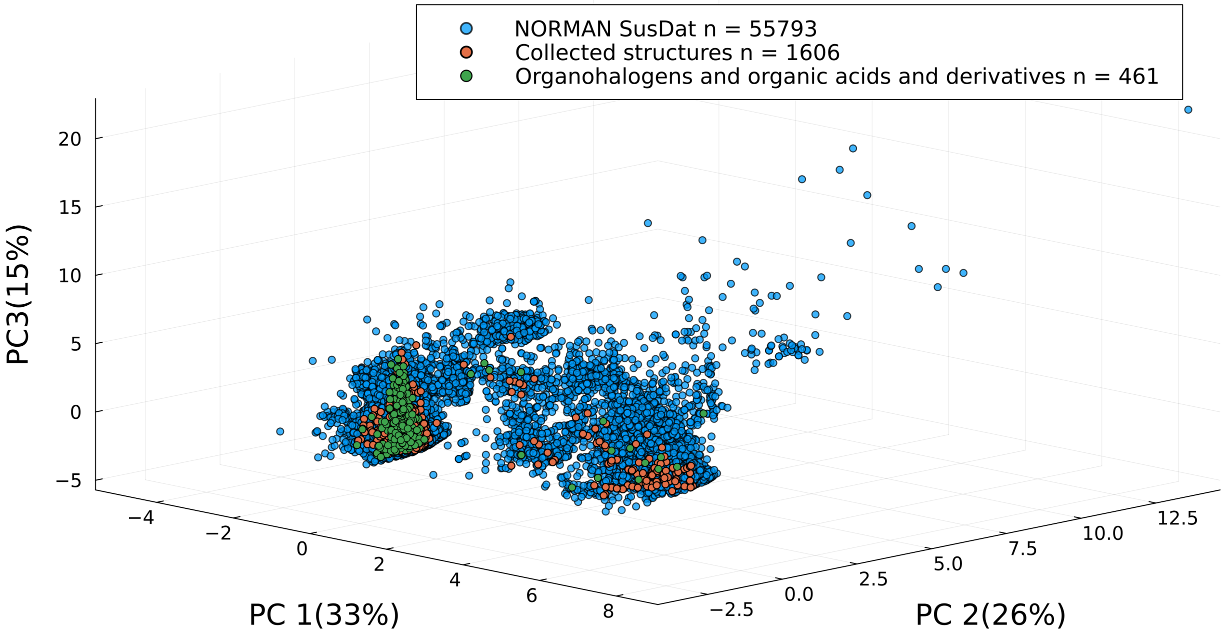

Figure S11: PCA of detected CECs and NORMAN SusDat chemicals with the organohalogen compounds and organic acids and derivatives highlighted
